# Dog Waste Creates a Preference‐Performance Mismatch for Urban Dung Beetles

**DOI:** 10.1002/ece3.74192

**Published:** 2026-08-12

**Authors:** Erin L. McCullough, Beatrice M. Altopp, Daniel F. Kellogg, Madelyn S. McInnis, Luna J. Pagan

**Affiliations:** ^1^ Department of Biology Clark University Worcester Massachusetts USA

**Keywords:** diet preference, dung quality, maternal provisioning, *Onthophagus orpheus*

## Abstract

Urban environments expose animals to novel conditions that may favor behavioral flexibility but can create preference‐performance mismatches when attractive resources are associated with reduced reproductive success. Dung beetles are often considered dietary generalists, yet little is known about how they respond to altered food availability in human‐dominated habitats. We combined field surveys and laboratory experiments using two domestic dung types to examine diet preference, maternal provisioning, and offspring performance in the urban dung beetle *Onthophagus orpheus* . Beetles strongly preferred dog dung over cow dung, and offspring reared on dog dung developed faster and emerged as larger adults. Despite these apparent benefits, brood ball mortality was more than twice as high on dog dung, with nearly 60% of brood balls failing compared to only 25% on cow dung. Thus, the dung type most strongly preferred by adults was also associated with the lowest offspring survival. This preference–performance mismatch suggests that accumulation of domestic dog waste—a pervasive feature of urban habitats—may pose a widespread but underappreciated risk to urban dung beetles.

## Graphical abstract

This study demonstrates a preference‐performance mismatch associated with an abundant urban resource. The dung beetle *Onthophagus orpheus* strongly prefers domestic dog dung despite substantially lower offspring survival, suggesting that the accumulation of pet waste in urban environments may have unintended ecological consequences for dung beetle populations.

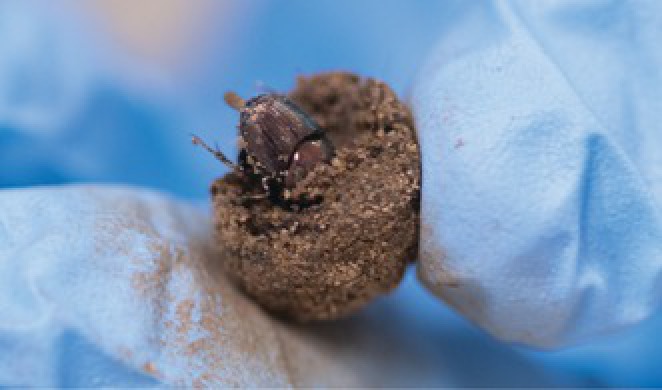

## 1 Introduction

Urbanization is one of the most rapid and dramatic forms of environmental change, exposing animals to novel resources and altered ecological conditions (Alberti et al. 2020). Behavioral plasticity can help individuals cope with such variability and may explain why some species survive or even thrive in human‐altered environments whereas others fail (Sih et al. 2011; Wong and Candolin 2015). However, behavioral plasticity can also increase susceptibility to evolutionary traps, in which animals preferentially exploit resources (e.g., food, oviposition sites) that ultimately reduce fitness (Robertson et al. 2013; Schlaepfer et al. 2002; Sih et al. 2011).

Dung beetles (Coleoptera: Scarabaeidae) are widely used as bioindicators of ecosystem health because they are highly sensitive to environmental disturbance (Davis et al. 2001; Nichols et al. 2007; Salomão et al. 2019). Because they primarily feed and breed in mammal dung, dung beetle and mammal communities are tightly linked (Bogoni et al. 2019; Raine and Slade 2019). Urbanization often reduces mammal diversity (Hansen et al. 2024), leading to declines in dung beetle abundance and species richness (Correa et al. 2021; Salomão et al. 2019). At the same time, urbanization introduces anthropogenic food resources, such as domestic dog waste, that may differ substantially from the native dung resources historically encountered by beetles (Carpaneto et al. 2005; Ramírez‐Restrepo and Halffter 2016).

Although most dung beetle species are classified as generalist feeders (Hanski and Cambefort 2014; Raine and Slade 2019), numerous studies have shown that they exhibit consistent preferences among dung types (Bogoni and Hernández 2014; Raine et al. 2019). Dung quality also strongly influences maternal provisioning, offspring development, and adult phenotype (Arellano et al. 2015; Hunt and Simmons 2000; Moczek 1998; Yap et al. 2024). Despite this sensitivity to dung type and quality, how dung beetles respond to altered food availability in urban environments remains poorly understood (Ramírez‐Restrepo and Halffter 2016).

Here, we investigate diet preference and offspring performance in the urban dung beetle *Onthophagus orpheus* , the most abundant dung beetle in urban forests in central Massachusetts (McCullough et al. 2025). Females dig tunnels beneath fresh dung pads and provision offspring by constructing brood balls in which they lay a single egg (Halffter and Edmonds 1982). We compared dung attractiveness, maternal provisioning, and offspring performance using cow and dog dung—two domestic dung types from distinct trophic guilds. Cow (herbivore) dung is a standard bait in dung beetle studies (Frank et al. 2018), whereas dog (omnivore) dung is the most abundant dung type in many urban habitats (Carpaneto et al. 2005; Ramírez‐Restrepo and Halffter 2016). We find that *O. orpheus* strongly prefers dog dung, but offspring reared on dog dung suffer high brood mortality, consistent with an evolutionary trap driven by an abundant urban resource.

## 2 Methods

We collected adult *Onthophagus orpheus* using live pitfall traps at two urban forests in Worcester, Massachusetts: Hadwen Arboretum (42°15′29″ N, 71°49′57″ W) and Cascades Park (42°17′17″ N, 71°51′55″ W). Pitfall traps consisted of a 1 L Nalgene bottle that was buried flush with the soil surface, covered with 0.75‐in plastic mesh, and secured with landscape staples. Each trap was baited with 90 mL of either cow or dog dung placed on top of the mesh. Dung baits were kept frozen until use. Eight traps (four per dung type) were deployed at each site twice per month from April to November 2024. Traps were spaced ≥ 10 m apart and left in place for 24 h before collection.

Because field collections from 2024 suggested a seasonal shift in diet preference, we conducted additional sampling from June to August 2025 to determine whether diet preference covaried with body size, and a laboratory rearing experiment to quantify the effects of dung type on maternal provisioning and offspring performance. Beetles were collected using live pitfall traps as described above and returned to the laboratory to be identified and measured (see McCullough et al. 2025 for details). Individuals were photographed in the dorsal position with a Leica K5C camera attached to a Leica M125C microscope, and prothorax width was measured as a proxy for body size using Leica Application Suite X image analysis software.

**FIGURE 1:**
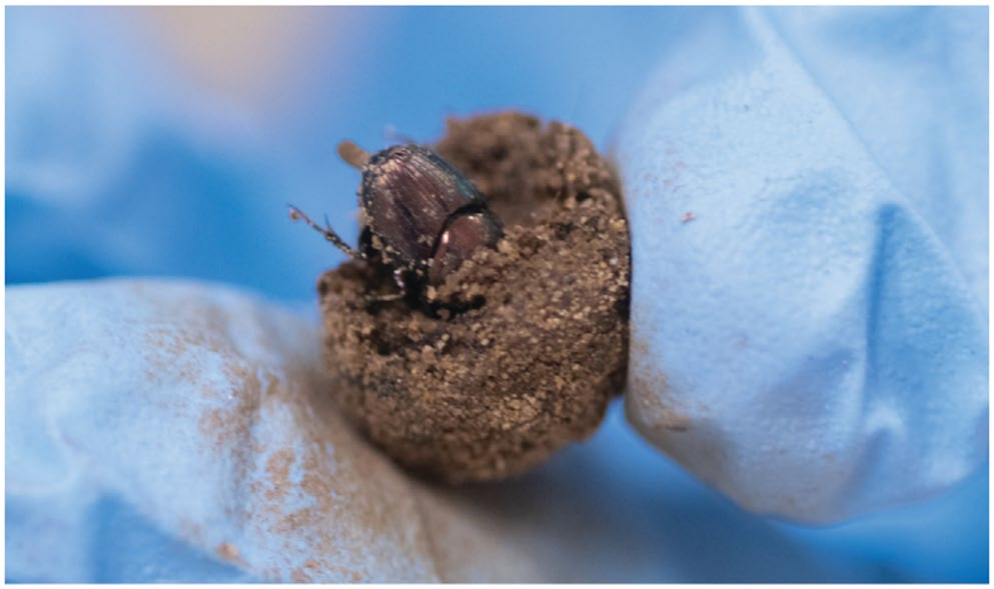
A female *Onthophagus orpheus* constructs a brood ball out of dog dung where she will lay a single egg. (Photo by Steven King).

After measurements, females were set up in individual breeding chambers to produce brood balls (Figure 1). Chambers consisted of PVC piping (25 cm length, 6 cm diameter) filled three‐quarters with a moist sand‐soil mixture and topped with 90 mL fresh dung. Half the females were provided with cow dung, and half were provided with dog dung. Chambers were maintained at room temperature (approximately 22°C) for 7 days and then sifted to recover brood balls and females. Each brood ball was cleaned of sand, weighed to the nearest 0.01 g with a digital balance (Mettler Toledo PL1502E), and buried in an individual plastic container (3 cm height, 6 cm diameter) filled with moist sand until emergence as adults. Females that produced brood balls were established in new breeding chambers with the alternate dung type for another 7 days before being sifted again for brood balls. Brood ball containers were checked daily for signs of emergence, and development time was recorded as the number of days between breeding chamber establishment and adult emergence. Newly emerged adults were sexed and measured for body size (prothorax width) as described above.

To minimize variation in dung quality, fresh cow dung for all experiments was collected from a single population of cattle in pastures in May 2023, homogenized, and frozen in clean plastic containers until use. Dog dung was collected throughout both seasons from a domestic dog fed a dry kibble diet. Fresh dog dung was collected in waste bags and frozen until use.

To test for significant diet preferences, we conducted binomial tests using monthly collection data from May to September 2024, when adult beetles were active. We conducted Welch's *t*‐tests to compare the body size of beetles attracted to cow versus dog dung. To test the effect of dung type on reproductive output, we fitted a linear mixed‐effects model (LMM) with brood number as the response variable, and dung type and female body size as fixed effects. Female ID was included as a random effect to account for non‐independence of brood balls from the same mother. To test the effect of dung type on maternal provisioning, we fitted a LMM with brood ball mass as the response variable, dung type, offspring sex, and female body size as fixed effects, and female ID as a random effect. To test the effect of dung type on offspring body size and development time, we fitted LMMs with either prothorax width or development time as the response variable, brood ball mass, dung type, and offspring sex as fixed effects, and female ID as a random effect. LMMs were conducted using the *lmer* function in the R package *lme4* (Bates et al. 2015). Models were initially fitted with all interaction terms and then simplified to the minimum adequate model by sequentially removing non‐significant terms using deletion tests (Crawley 2012). Finally, we conducted chi‐square tests to compare the effect of dung type on mortality rate (i.e., proportion of dead brood) and secondary sex ratio (i.e., proportion of males among emerged beetles). All analyzes were conducted in R version 2025.09.2 + 418 (R Core Team 2025).

## 3 Results

**FIGURE 2:**
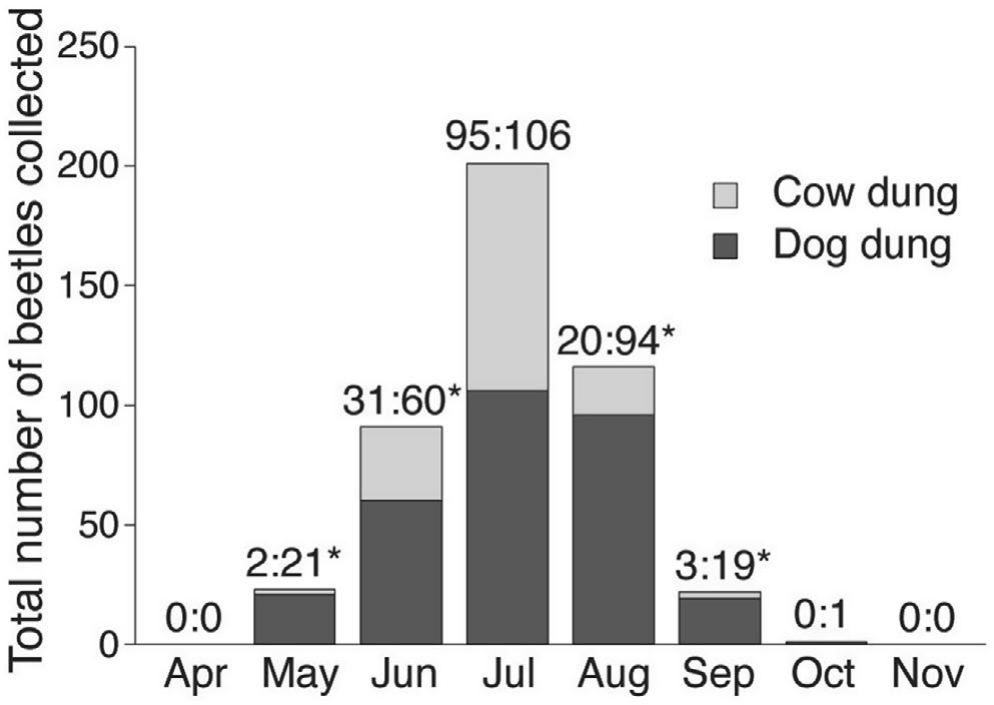
Stacked bar plot showing seasonal activity and diet preference. Bar heights and reported ratios (cow:dog) represent the total number of beetles collected each month, with beetles collected on cow dung represented in light gray and beetles collected on dog dung represented in dark gray. Asterisks denote significant diet preference (binomial test: *p* < 0.01).

*Onthophagus orpheus* had a strong preference for dog dung over cow dung, with 67% of individuals being attracted to pitfall traps baited with dog dung across the 2024 field season (Figure 2). We collected a total of 452 individuals (174 males, 278 females). Beetles preferred dog dung over cow dung in May, June, August, and September (binomial tests: *p* < 0.01), but showed no preference in July (binomial test: *p* = 0.48), when beetles were most abundant. Diet preference did not differ between sexes (*χ* ^2^ < 0.001, df = 1, *p* = 1), and there was no difference in body size between beetles attracted to cow versus dog dung (*t* = 0.14, df = 134.58, *p* = 0.89).

**FIGURE 3:**
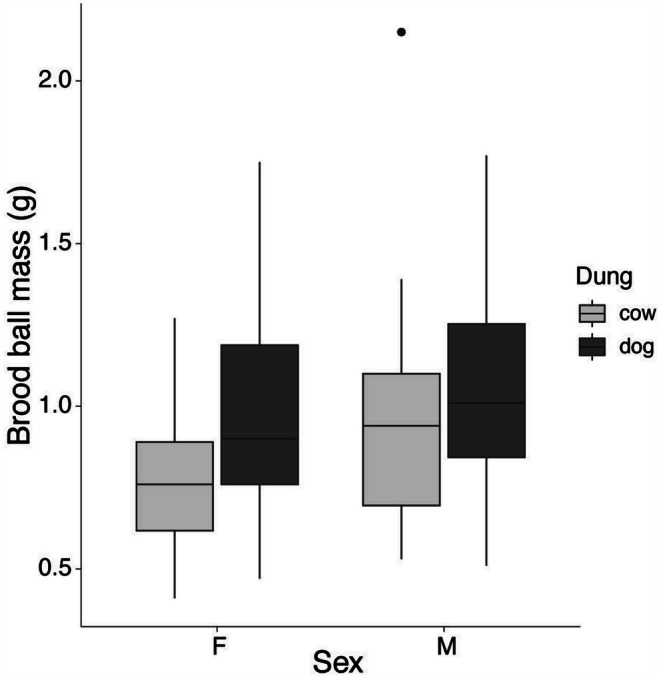
Boxplot showing the effect of dung type, offspring sex, and their interaction on brood ball mass. Brood balls were larger on dog dung compared to cow dung (*β* = 0.24, *t* = 4.18, *p* < 0.001); male offspring developed in larger brood balls than female offspring (*β* = 0.18, *t* = 2.23, *p* = 0.03); and the sex difference in brood ball mass was more pronounced on cow dung than dog dung (*β* = −0.21, *t* = −2.06, *p* = 0.04).

Reproductive output was not influenced by female size (*β* = 0.76, *t* = 0.92, *p* = 0.37) or dung type (*β* = −0.39, *t* = −1.13, *p* = 0.27). Brood ball mass was significantly influenced by dung type (*β* = 0.24, *t* = 4.18, *p* < 0.001), offspring sex (*β* = 0.18, *t* = 2.23, *p* = 0.03), and their interaction (*β* = −0.21, *t* = −2.06, *p* = 0.04; Figure 3). Brood balls were larger on dog dung compared to cow dung; male offspring developed in larger brood balls than female offspring, and the sex difference in brood ball mass was more pronounced on cow dung than dog dung.

**FIGURE 4:**
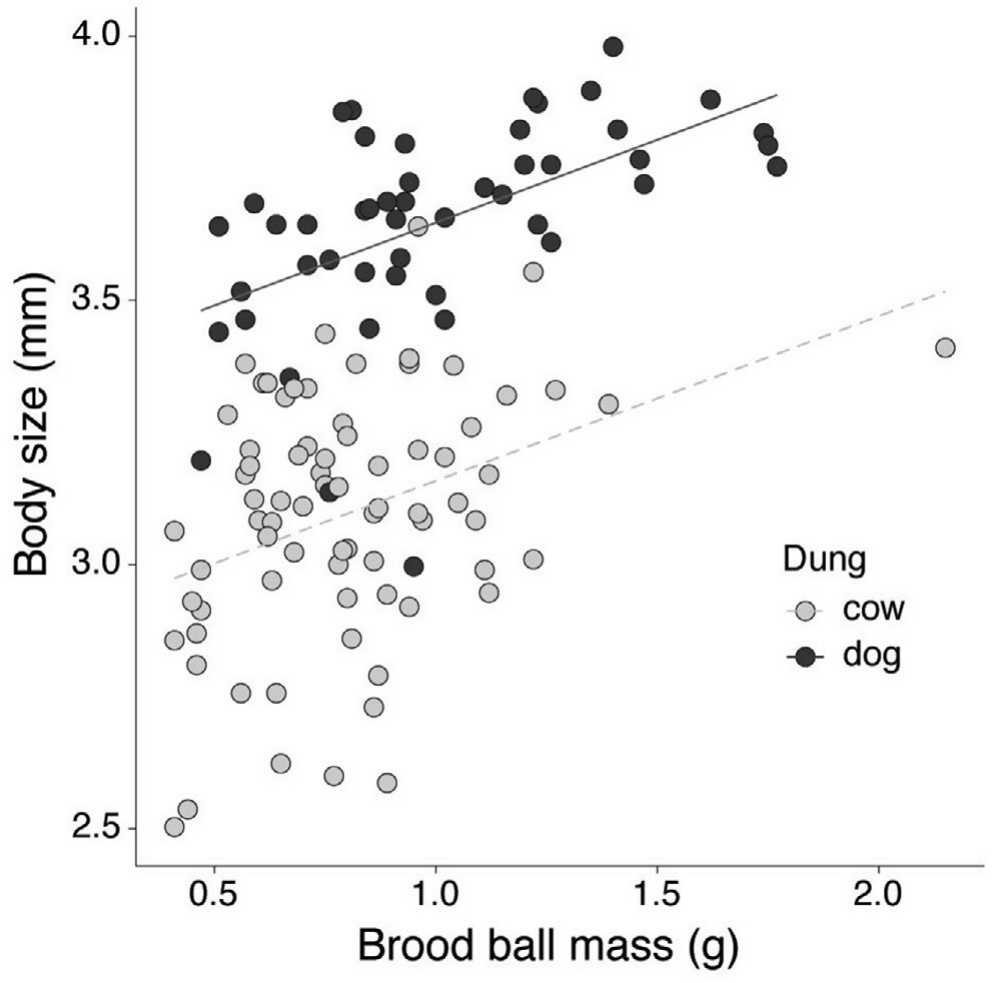
Scatterplot showing the effect of dung type and brood ball mass on adult body size. Larger brood balls produced larger adults (*β* = 0.31, *t* = 4.75, *p* < 0.001), and at any given brood ball mass, beetles reared on dog dung were larger those reared on cow dung (*β* = 0.49, *t* = 12.46, *p* < 0.001).

Adult body size was significantly influenced by brood ball mass (*β* = 0.31, *t* = 4.75, *p* < 0.001) and dung type (*β* = 0.49, *t* = 12.46, *p* < 0.001; Figure 4). Larger brood balls produced larger adults, and at any given brood ball mass, beetles reared on dog dung were larger than those reared on cow dung.

**FIGURE 5:**
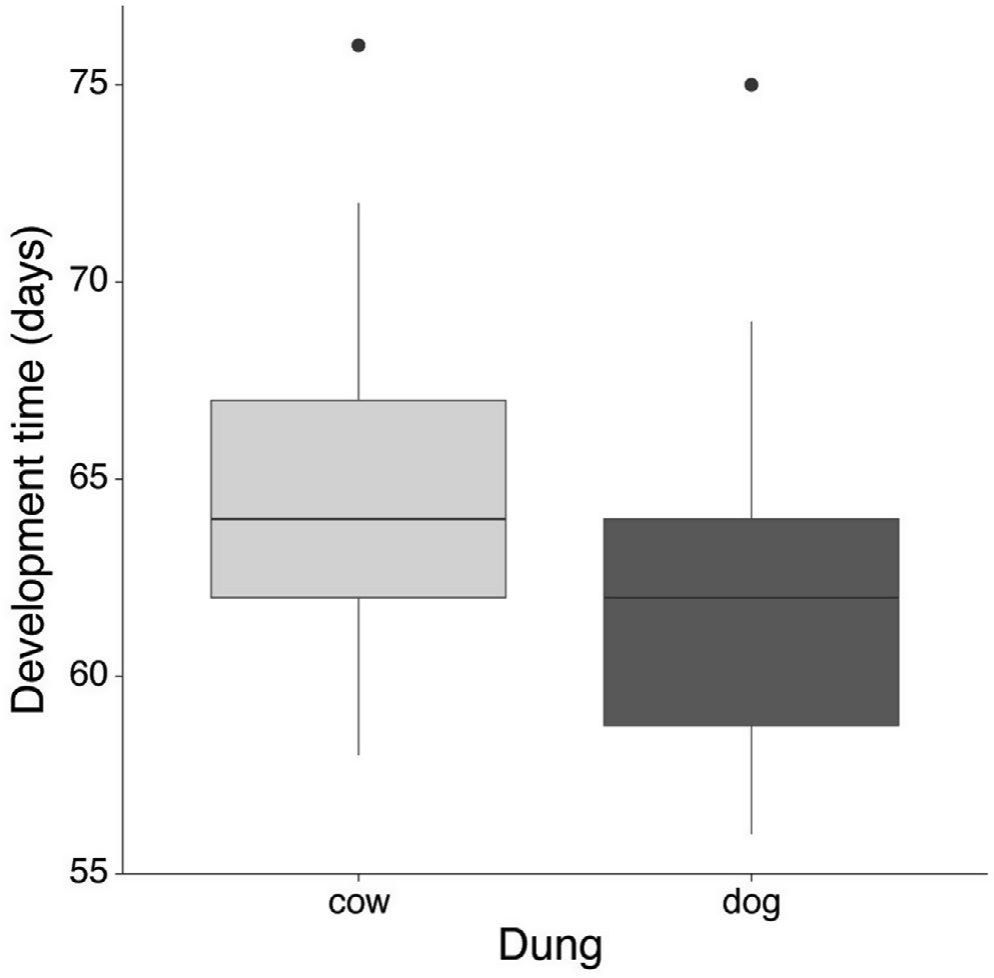
Boxplot showing the development time of beetles reared on cow dung and dog dung. Beetles developed significantly faster on dog dung compared to cow dung (*β* = −2.22, *t* = −3.28, *p* = 0.001).

Development time was significantly influenced by dung type (*β* = −2.22, *t* = −3.28, *p* = 0.001), offspring sex (*β* = −2.21, *t* = −3.29, *p* = 0.001), and brood ball mass (*β* = 3.84, *t* = 3.43, *p* < 0.001). Beetles developed faster on dog dung compared to cow dung; male offspring developed faster than female offspring; and larger brood balls had longer development times than smaller brood balls. On average, beetles took 62.0 days to complete development on dog dung versus 64.6 days on cow dung (Figure 5).

Brood balls that failed during development did not differ in mass from those that successfully produced adults (*t* = −0.89, df = 200.17, *p* = 0.38), but mortality was significantly higher on dog dung compared to cow dung (*χ* ^2^ = 24.56, df = 1, *p* < 0.001). The mortality rate was 59.3% on dog dung versus 25.0% on cow dung.

The secondary sex ratio (SSR) differed between dung types (*χ* ^2^ = 19.88, df = 1, *p* < 0.001). On cow dung, the sex ratio was significantly female‐biased (11 males of 75 offspring; SSR = 0.15; binomial test: *p* < 0.001), whereas on dog dung, the sex ratio did not differ from parity (26 males of 48 emerged offspring; SSR = 0.54; binomial test: *p* = 0.67).

## 4 Discussion

Our study demonstrates that the urban dung beetle *Onthophagus orpheus* exhibits strong diet preferences across its breeding season, and that dung type significantly influences maternal provisioning and offspring performance. Beetles consistently preferred dog dung over cow dung, but offspring suffered significantly higher mortality when reared on the preferred dung type. These results demonstrate a preference–performance mismatch that may represent an evolutionary trap for urban dung beetles given the increased availability of dog dung in urban environments (Ramírez‐Restrepo and Halffter 2016; Robertson et al. 2013; Steward and Boggs 2020).

Beetles strongly preferred dog dung over cow dung in four of the five months in which adults were active but showed no dietary preference when activity peaked in July. These results indicate that dog dung is generally a highly attractive resource for *O. orpheus* , although diet preferences may shift seasonally as environmental conditions change. In particular, higher temperatures in July may drive beetles to favor cow dung because its higher moisture content makes it a more suitable resource under hot, drying conditions (Wuthisuthimethavee et al. 2024). We found no difference in body size between beetles attracted to dog versus cow dung, which suggests that larger individuals do not monopolize the preferred dung type and that dietary requirements are not size‐dependent. We note that both cow and dog dung represent exotic resources for *O. orpheus* that are likely to differ in nutritional composition from the native dung sources to which this species evolved. Although the native dung sources for *O. orpheus* are unknown, wildlife sightings at our study sites suggest that they include dung from both herbivores (e.g., white‐tailed deer) and omnivores (e.g., coyotes; see also Stone et al. 2025).

Previous studies have shown that *Onthophagus* females strategically adjust their provisioning in response to dung quality, by producing more but smaller brood masses on high‐quality dung (Arellano et al. 2015; Hunt and Simmons 2004; Kishi and Nishida 2006). Dung quality also affects offspring performance, with beetles developing more quickly and emerging as larger adults when reared on higher‐quality dung (Arellano et al. 2015; Kishi and Nishida 2006; Moczek 1998; Yap et al. 2024). Although the specific sources of high‐quality dung differed among studies, omnivore dung was identified as higher quality than herbivore dung in multiple cases (Kishi and Nishida 2006; Yap et al. 2024). Consistent with these patterns, we found that *O. orpheus* developed faster and achieved larger adult body sizes on dog (omnivore) dung than on cow (herbivore) dung, which suggests that dog dung is the higher‐quality resource. However, these benefits were offset by substantially higher mortality: nearly 60% of brood balls failed on dog dung compared to only 25% on cow dung. Females also did not adjust their provisioning in the predicted direction; there was no difference in brood ball number between dung types, and females produced larger brood balls on dog dung, the preferred dung type. These results demonstrate that adult preference and enhanced offspring growth do not necessarily translate into higher reproductive success, highlighting a clear preference‐performance mismatch.

The mechanism underlying the higher mortality observed on dog dung remains unresolved. Dog dung and cow dung differ in their moisture content, nutrient composition, microbial communities, and potential presence of parasites, pharmaceutical residues, and microplastics (McConnell et al. 2026; Mutungwazi et al. 2022; Ramírez‐Restrepo and Halffter 2016; Wuthisuthimethavee et al. 2024). Any of these factors could have contributed to the reduced offspring survival observed on dog dung. Exposure to veterinary pharmaceuticals is an intriguing possibility because macrocyclic lactones (e.g., ivermectin), which are administered to livestock and excreted in feces, are known to increase larval and pupal mortality in dung‐dwelling insects (Floate et al. 2005; Jacobs and Scholtz 2015; Lumaret et al. 2012). Most domestic dogs are routinely treated with antiparasitic medications that contain macrocyclic lactones (Massetti et al. 2023; Mwacalimba et al. 2023; Pennelegion et al. 2020), making veterinary pharmaceuticals a plausible factor contributing to the high brood ball mortality on dog dung. Another possibility is exposure to microplastics, which have been shown to reduce survival in dung beetle larvae (McConnell et al. 2026) and are commonly found in animals from urban settings (Prata et al. 2022; Wayman et al. 2024). Future experiments are needed to determine the relative contributions of these factors to the mortality patterns observed here.

Dung type also affected the secondary sex ratio of beetles reared in the laboratory. Cow dung yielded a strongly female‐biased sex ratio, whereas dog dung yielded an approximately equal sex ratio. Notably, the secondary sex ratio of beetles reared on cow dung was more than twice as female‐biased as the adult sex ratio observed for *O. orpheus* in the field (SSR = 0.15 vs. ASR = 0.38; McCullough et al. 2025). Male offspring emerged from heavier brood balls than female offspring, which aligns with previous findings that *Onthophagus* females provision more resources to sons than daughters (House et al. 2011). Although these findings suggest that *O. orpheus* females could influence sex allocation through differential provisioning, we predict that the differences in sex ratio observed between cow and dog dung are driven primarily by sex‐biased mortality rather than adaptive sex allocation. If dog dung is more nutritionally stressful than cow dung, and if females are more sensitive to nutritional stress, then higher female mortality on dog dung could provide a non‐adaptive explanation for the different sex ratios observed in our rearing experiment (House et al. 2011; Quezada‐García et al. 2014).

Overall, this study demonstrates how an abundant anthropogenic resource—domestic dog waste—can create a preference‐performance mismatch for urban dung beetles. Dog dung is consistently preferred by *O. orpheus* adults despite being associated with markedly lower offspring survival. Our findings suggest that the accumulation of pet waste in urban greenspaces may pose a widespread but underappreciated risk to urban dung beetles, with potential consequences for dung beetle communities and the ecosystem functions they provide.

## Author Contributions

**Erin L. McCullough:** conceptualization (lead), formal analysis (lead), investigation (equal), visualization (lead), writing – original draft (lead), writing – review and editing (lead). **Beatrice M. Altopp:** investigation (equal), writing – review and editing (supporting). **Daniel F. Kellogg:** investigation (equal), writing – review and editing (supporting). **Madelyn S. McInnis:** investigation (equal), writing – review and editing (supporting). **Luna J. Pagan:** investigation (equal), writing – review and editing (supporting).

## Conflicts of Interest

The authors declare no conflicts of interest.

## Data Availability

All data and code for the analyses reported in this article are available on Dryad: https://doi.org/10.5061/dryad.1jwstqk8q
